# *KAT2A*/*E2F1* Promotes Cell Proliferation and Migration via Upregulating the Expression of *UBE2C* in Pan-Cancer

**DOI:** 10.3390/genes13101817

**Published:** 2022-10-08

**Authors:** Shudai Lin, Li Qiu, Keying Liang, Haibo Zhang, Mingjian Xian, Zixi Chen, Jinfen Wei, Shuying Fu, Xiaocheng Gong, Ke Ding, Zihao Zhang, Bowen Hu, Xiquan Zhang, Yuyou Duan, Hongli Du

**Affiliations:** 1 School of Biology and Biological Engineering, South China University of Technology, Guangzhou 510006, China; 2 College of Coastal Agricultural Sciences, Guangdong Ocean University, Zhanjiang 524088, China; 3 College of Animal Science, South China Agricultural University, Guangzhou 510642, China; 4 Laboratory of Stem Cells and Translational Medicine, Institutes for Life Sciences and School of Medicine, South China University of Technology, Guangzhou 510006, China

**Keywords:** lysine acetyltransferase 2A (*KAT2A*), E2F transcription factor 1 (*E2F1*), ubiquitin conjugating enzyme E2 C (*UBE2C*), cell proliferation, cell migration, cell cycle, pan-cancer

## Abstract

Various studies have shown that lysine acetyltransferase 2A (*KAT2A*), E2F transcription factor 1 (*E2F1*), and ubiquitin conjugating enzyme E2 C (*UBE2C*) genes regulated the proliferation and migration of tumor cells through regulating the cell cycle. However, there is a lack of in-depth and systematic research on their mechanisms of action. This study analyzed The Cancer Genome Atlas (TCGA) to screen potential candidate genes and the regulation network of *KAT2A* and *E2F1* complex in pan-cancer. Quantitative real-time PCR (qRT-PCR) and Western blotting (WB), cell phenotype detection, immunofluorescence co-localization, chromatin immunoprecipitation assay (ChIP), and RNA-Seq techniques were used to explore the functional of a candidate gene, *UBE2C*. We found that the expression of these three genes was significantly higher in more than 10 tumor types compared to normal tissue. Moreover, *UBE2C* was mainly expressed in tumor cells, which highlighted the impacts of *UBE2C* as a specific therapeutic strategy. Moreover, *KAT2A* and *E2F1* could promote cell proliferation and the migration of cancer cells by enhancing the expression of *UBE2C*. Mechanically, *KAT2A* was found to cooperate with *E2F1* and be recruited by *E2F1* to the *UBE2C* promoter for elevating the expression of *UBE2C* by increasing the acetylation level of H3K9.

## 1. Introduction

Many cancer treatment strategies have been developed based on targeting specific molecules related to specific gene mutation or specific gene expression, and breaking progress is being made with these cancer treatment strategies, and the potential is infinite [1,2,3]. Therefore, there is an urgent need to identify efficient therapeutical targets in tumors. Currently, many common molecular mechanisms across pan-cancer have been discovered by various studies [4,5,6]. High-throughput transcriptome was an important and effective data to identify the candidate targets or pathways [7,8,9]. Although tumors have great heterogeneity, there are commonalities among different types of tumors, so using the transcriptome data of large clinical pan-cancer samples to authenticate potential candidate targets upstream may be a good option. However, whether it will shed light on future cancer treatment still needs further comprehensive and in-depth research on pan-cancer.

Previous studies have shown that there were abnormalities of histone acetylation modification in various cancers, including liver, lung, and breast [10,11]. Histone acetylation mediates the expression and activation of genes related to cell proliferation, differentiation, and apoptosis, which could affect the occurrence and development of tumors [12,13] Histone lysine acetyltransferases (KATs) and histone deacetylases (HDACs) are key effectors balancing between histone acetylation and deacetylation. *KAT2A*, the first discovered KAT that was related to transcription, has been reported to be involved in gene transcription, DNA repair, nucleosome assembly, and cell cycle regulation in pan-cancer. Additionally, it was significantly up-regulated in many cancers to promote the growth of tumor cells/cell proliferation, and the invasion and migration of cancer cells [14,15,16,17,18,19,20]. Therefore, *KAT2A* may be a significant oncological target with effective therapeutics for several cancers.

*E2F1* was found as a key regulator of G1/S transition, and to promote the transcription of plenty of critical genes for cell-cycle progression [21]. Many reports have found that *E2F1* played a central role in cancer development, such as in breast cancer [22,23], bladder cancer [24], and prostate cancer [25]. It is suggested that the up-regulation of *E2F1* can promote the proliferation, migration, and invasion of these cancer cells, and it is also significantly related to the clinical stage of different cancer types, the depth of tumor invasion, as well as the metastasis and lesion size of lymph nodes [26]. As a crucial catalytic component of transcription regulation complex, it has been suggested that *KAT2A* can increase the chromatin accessibility of transcription factors (such as *E2F1*) and form protein complexes with them. Moreover, it could be recruited to the promoter regions of genes involved in the cell cycle, DNA damage repair, and cell migration, consequently enhancing their expression through increasing the acetylation level of H3K9 on these gene-promoting regions [27,28]. For example, *KAT2A* has been explored to cooperate with *E2F1* and be recruited by *E2F1* to the promoters of cyclin D1 and cyclin E1 [16], and it is amplified in breast cancer 1 genes [29].

*UBE2C* is a member of the E2 ubiquitin-conjugating enzyme family [30]. Ubiquitination is an important cellular mechanism for targeting proteins for degradation, which is involved in numerous cell processes, such as cell cycle progression, antigen presentation, transcription, and programmed cell death [31]. Many studies have shown that the expression of UBE2C is upregulated in a variety of human malignancies, such as tongue squamous cell carcinoma [32], breast cancer [33], endometrial cancer [34], melanoma [35], and rectal carcinoma [36]. All these findings suggested that UBE2C is closely associated with the development of cancer, and could be used as a potential therapeutic target for different types of cancers.

Taken together, these reports indicated that *KAT2A*, *E2F1*, and *UBE2C* play a fundamental role in the progression of several types of cancers. Hitherto, no conclusive study has reported the role of *KAT2A* and *E2F1* interactions in the pan-cancer landscape. With such conspicuous roles of both *KAT2A* and *E2F1* in cellular functions and putative links to cancer, we investigated the common molecular mechanisms and potential transcription target of their interaction across pan-cancer. This study revealed the common characteristics of *KAT2A*/*E2F1*/*UBE2C* and clarified the mechanism of this axis across pan-cancer through RNA-seq dada and in vitro experiments, which might shed light on pan-cancer treatment.

## 2. Materials and Methods

### 2.1. Data Source

The expression data and corresponding clinical information of different kinds of cancer patients were downloaded from The Cancer Genome Atlas (TCGA). The GSE137172 set was downloaded from the Gene Expression Omnibus database (GEO; http://www.ncbi.nlm.nih.gov/geo/, accessed on 13 January 2021) for analyzing differentially expressed genes (DEGs) after knocking down *UBE2C*.

### 2.2. Transcriptional Expression of Different Genes in Pan-Cancer

*KAT2A*, *E2F1*, and *UBE2C* were formed with the Transcripts Per Million (TPM) mean of each mRNA expression of samples in TCGA, and the exact sample sizes of cancer and normal samples used are reported in Table S1. The expression profiles of *KAT2A*, *E2F1*, and *UBE2C* were analyzed using GEPIA (Gene Expression Profiling Interactive Analysis, http://gepia.cancer-pku.cn/, accessed on 14 April 2021) online analysis [37]. Then, comparisons between tumor and normal tissues were analyzed. The relative expression levels of *KAT2A*, *E2F1*, and *UBE2C* to *ACTB* were also analyzed using corresponding cancer cell lines in the Cancer Cell Line Encyclopedia (CCLE) database, respectively.

### 2.3. Pathological Staging Expression Analysis

The TPM expression of *KAT2A*, *E2F1*, and *UBE2C* at different pathological stages in the TCGA database were represented by box plots, and Student’s *t*-test was employed to compare the relative expression levels among different pathological stages. *p* < 0.05 indicated statistically significant differences.

### 2.4. Prognostic Analysis of Patients in Pan-Cancer

The clinical outcome of patients with different types of cancers was determined using Kaplan–Meier survival curves. For the overall survival (OS), the samples were divided into two groups according to the median expression of the mRNAs (high vs. low). With the use of R packages (survival, version 3.2.7; survminer, version 0.4.8), Kaplan–Meier survival analysis and the log-rank test were employed to compare OS between the tumor and normal cohorts. *p* < 0.05 indicated statistically significant differences.

### 2.5. Identification of Differentially Expressed Genes of Pan-Cancer from TCGA

The samples of different types of cancers in the TCGA databases were separated into 30% each of *KAT2A*, *E2F1*, and *UBE2C* high and low groups to obtain DEGs using the “DESeq2” package (version 1.28.1) in R language (version 4.0.2). |Fold Change| > 1.5 and FDR < 0.05 were set as the statistical threshold value of DEGs. Using the transcriptome data of 11 tumor types (with normal tissues more than 30) in TCGA, which contains the significantly highly expressed level of *KAT2A*, overlapping DEGs were screened according to the following conditions: each tumor *KAT2A* and *E2F1* were grouped according to the 30% high and low groups to obtain: the DEGs (FC > 1.2, FDR < 0.05, *KAT2A* 30%-Up, and *E2F1* 30%-Up), respectively; the correlation coefficient with *KAT2A* and *E2F1* > 0.3, respectively; and DEGs that were highly expressed in tumor tissues compared with normal tissues.

### 2.6. Spearman Correlation among KAT2A, E2F1, and UBE2C

Spearman’s correlation coefficient analysis was performed to explore the correlation among *KAT2A*, *E2F1*, and *UBE2C* in 11 out of 33 cancers with more than 200 tumor tissues from the TCGA database, and the corresponding cell lines in the CCLE database.

### 2.7. Functional Enrichment Analysis

Functional enrichment analysis, gene ontology (GO), and Kyoto Encyclopedia of Genes and Genomes (KEGG) analyses were conducted by the R package (clusterProfiler, version 3.16.1) to explore the different molecular mechanisms and involved pathways between high and low *UBE2C* expression. The protein–protein interaction (PPI) network of DEGs was obtained from the STRING (version 11.0) database [38].

### 2.8. Analyzing the Candidate Genes Regulated by UBE2C and Related to Tumor Cell Proliferation, Cell Cycle, and Apoptosis Based on the Transcriptome Data of the TCGA and GEO Dataset

The results from the TCGA RNA transcriptome data and the existing GEO dataset were combined to obtain the overlapping DEGs, and the key genes or target proteins and signal pathways regulated by *UBE2C* were searched. The common mechanism of *KAT2A*/*E2F1*/*UBE2C* affecting tumor cell proliferation, cell cycle, and apoptosis in different tumors were explored through GO and KEGG function enrichment analyses.

### 2.9. Single-Cell RNA-Seq Data Processing

The single-cell RNA-Seq data were analyzed as described previously [39] Expression data were extracted from a previous study [39] and violin plots were drawn using R.

### 2.10. Cell Lines and Transfection

Parental MCF-7 breast cancer, NCI-H460 large cell lung carcinoma HepG2 liver cancer, and BxPC3 pancreatic cancer cell lines were gifts from Dr. Peng Huang, Sun Yat-sen University Cancer Hospital, Guangzhou, China. The 786-O renal clear cell carcinoma cell line was purchased from Cell Resource Center, Shanghai Academy of Biological Sciences, Chinese Academy of Sciences. MCF-7, HepG2, and BxPC3 were cultured in DMEM medium with 10% fetal bovine serum, penicillin (100 U/mL), and streptomycin (100 U/mL) at 37 °C in air with 5% CO_2_. MCF-7 was cultured with 0.2 mg/mL insulin. NCI-H460 and 786-O were in RPIM-1640 medium with 10% fetal bovine serum, penicillin (100 U/mL), and streptomycin (100 U/mL) at 37 °C in air with 5% CO_2_. For transient knockdown studies, KAT2A-shRNA, UBE2C-shRNA (Fitgene, Guangzhou, China), and control shRNA (shNC) plasmids, and a final concentration of 60 nM of both E2F1-siRNA and control siRNA (siNC) (Hanheng, Shanghai, China) (Table S2) were transfected for 24 h according to Lipofectamine™ 3000 (Thermo Fisher Scientific, Waltham, MA, USA). NCI-H460 cells with a stable knockdown of *KAT2A* were established by transfection with a KAT2A-shRNA (shKAT2A-1) lentiviral vector.

### 2.11. Real-Time Quantitative Polymerase Chain Reaction (qPCR)

Real-time quantitative PCR (qPCR) analysis was performed according to the user’s manual using the StepOnePlus™ Real-Time PCR System (Applied Biosystems, Foster City, CA, USA) and Power SYBR Green PCR Master Mix (Applied Biosystems) kits. All samples were analyzed in triplicate, and the expression of *KAT2A, E2F1,* and *UBE2C* was normalized relative to that of *GAPDH*, which was used as an internal loading control. The primers for qPCR are listed in Table S2.

### 2.12. Western Blotting and Antibodies

The whole-cell lysate or the immunocomplexes were separated by 8 to 12% SDS-PAGE and transferred onto a polyvinylidene difluoride (PVDF) membrane (Millipore, Billerica, MA, USA). Anti-KAT2A (Cell Signaling, Danvers, MA, USA, 1:1000, #3305), anti-E2F1 (Invitrogen, Waltham, MA, USA; Thermo Fisher Scientific, Waltham, MA, USA, 1:1000, MA1-23202), anti-H3K9ac (Cell Signaling Technology, 1:1000, 9649S), anti-UBE2C (Invitrogen, Thermo Fisher Scientific, 1:1000, PA5-27223), anti-β-actin (Beyotime Biotechnology, Shanghai, China, 1:1000, AA128), and horseradish peroxidase (HRP)-conjugated secondary antibodies (anti-mouse and anti-rabbit IgG) (Beyotime Biotechnology, 1:2000, A0208, A0216) antibodies were used to detect each protein. Bands were detected using BeyoECL Star chemiluminescence substrate (Beyotime Biotechnology, P0018AM).

### 2.13. Cellular Immunofluorescence

The detailed immunohistochemistry procedures were performed as described before [40]. Cells were seeded into the 35 mm laser confocal petri dishes under normal culture conditions to reach 60% density without any treatment to prepare for performing cell immunofluorescence. After incubating with *KAT2A* and *E2F1* primary antibodies (Invitrogen, Thermo Fisher Scientific, 1:1000, MA5-14884, MA1-23202), the cells were then incubated with the corresponding diluted IgG fluorescent secondary antibodies (Invitrogen, Thermo Fisher Scientific, 1:5000, A11029, A11012) for 1 h at room temperature in the dark. Then, the cells were stained with nucleus DAPI dropwise and incubated for 5 min in the dark. The mounting solution containing anti-fluorescence quencher (Invitrogen, Thermo Fisher Scientific, P36971) was dropped, followed by observing and collecting the image under a fluorescence microscope (Wetzlar, Germany, Leica TCS SP8 X).

### 2.14. Chromatin Immunoprecipitation (ChIP) and Co-Immunoprecipitation (Co-IP) Assay

ChIP was performed according to the instructions of the Pierce Agarose ChIP Kit (Thermo Fisher Scientific, 26156). The E2F1 binding sites on the *UBE2C* promoter were analyzed using the JASPAR online tool (http://jaspar.genereg.net/, accessed on 8 June 2020). ChIP-qPCR data were shown as the percentage of input following normalization with no antibody (mock). The primers for ChIP-qPCR are listed in Table S2. Co-IP was performed using indicated antibodies and IgG (Invitrogen) according to the manufacturer’s instructions. In brief, cell lysates were incubated with antibody-conjugated beads at 4 °C for 2 h. Then, the beads were washed extensively and boiled in SDS loading buffer. A total of 4% of total protein was used per IP, about 50 µg/100 µg.

### 2.15. Luciferase Assay

The luciferase assay was performed as described previously [41].

### 2.16. Cell Proliferation Assay

Cell proliferation assays were performed using the Cell Counting Kit-8 (CCK-8; Sangon Biotech, Shanghai, China) according to the manufacturer’s instructions. Briefly, cells were seeded onto 96-well plates (3 × 10^3^ cells per well) and transfected when they reached 70–80% of confluence according to the protocol of Lipofectamine™ 3000, and were then added with 10 µL of CCK-8 solution and cultured for 1 h at 37 °C in air with 5% CO_2_ on designated days. The absorbance was measured at 450 nm using TECAN infinite M200 (Softmax Pro., Molecular Devices, Sunnyvale, CA, USA). For EdU assay, the cells were treated for 48 h, followed by using the BeyoClick™ EdU Cell Proliferation Kit with Alexa Fluor 594 (Beyotime Biotechnology, C0078S) according to the manufacturer’s protocol.

### 2.17. Clone Formation Experiment

The stable *KAT2A* overexpression NCI-H460 cells were seeded in a 6-well plate with 2000 cells per well. After culturing for 14 days, the culture medium was discarded, was washed carefully with 1 × phosphate-buffered saline (PBS) (Gibco, Grand Island, NY, USA) once, and 1 mL of methanol solution was added to each well to fix the cells for 30 min. The methanol solution was aspirated, 1 mL of crystal violet stain solution was added to each well, and it was left at room temperature for 30 min. The crystal violet was recycled and each well was washed with distilled water, and the culture plate was placed upside down on absorbent paper to absorb the water. It was dried naturally, and pictures were taken using a digital camera. The number of clones with more than 10 cells under the microscope (4 × magnification) was counted. Finally, we calculated the clone formation rate = (number of clones/number of inoculated cells) × 100%.

### 2.18. Cell Migration and Invasion Assay

A wound healing assay was performed to detect the migration of three kinds of cancer cell lines after treatment. Cells growing the in log phase were trypsinized and seeded in 24-well plates until confluent. A total of 1 × 10^5^ cells per well were seeded in 24-well plates. After 24 h, the cells were transfected with shKAT2A-1, siE2F1-2, and shUBE2C-3, and the corresponding control shNC and siNC using Lipofectamine^TM^ 3000 (Thermo Fisher Scientific, Waltham, MA, USA) according to the manufacturer’s protocol. After transfection, cells were incubated at 37 °C and 95% confluent cells were used for a wound healing assay. Wounds were made using a 10 µL sterile tip. After incubation for 0, 24, 48, 72, and 96 h, the cells were photographed under an inverted microscope, respectively. The distance between the two edges of the scratch (wound width) was measured at 8 sites using ImageJ in each image (40× magnification). MCF-7, 786-O, and NCI-H460 cells were transiently transfected with shUBE2C-3 for 48 h, followed by using Falcon^®^ Permeable Support for a 24-well Plate with 8.0 µm Transparent PET Membrane (Corning, NY, USA) for measuring cell migration and invasion. After taking pictures of the cells, we added 1 mL absolute ethanol. With sufficient shaking, the light absorbance was measured at 570 nm. The optical absorbance (OD) value was used to plot the dilution of the samples, and the curves of the standard product and the samples were compared.

### 2.19. Cell Apoptosis Assay

The cells were treated with shUBE2C-3 and shNC for 48 h, respectively. Afterwards, the cells were digested with trypsin without EDTA. After termination, the cells were centrifuged at 1000 rpm for 5 min at 4 °C to remove the supernatant containing trypsin, and were then washed 2 times with precooled PBS. The cells were regenerated with serum-free medium, and cell apoptosis was analyzed using an Annexin V-FITC Apoptosis Detection Kit (Beyotime Biotechnology, C1062M) according to the instructions.

### 2.20. Cell Cycle Assay

After 48 h treatment of shNC and shUBE2C, the cells were washed twice with precooled PBS, centrifuged at 1200 rpm 4 °C for 5 min, fixed with 70% ethanol, and then stained with 10 µg/mL propidium iodide in a solution containing 100 µg/mL RNase in PBS; the cell pellet was slowly and fully resuspended, and was incubated at 37 °C for 30 min in the dark. Then, the cells were analyzed on a BD FACSCalibur (Becton, Dickinson and Company, San Jose, CA, USA).

### 2.21. RNA-Seq

Total RNA was extracted from the cells transfected for 48 h using Trizol reagent (Takara, Japan) and stored at −80 °C. The complementary DNA (cDNA) libraries of each pooled RNA sample for single-end sequencing were generated using the NEBNext^®^ UltraTM RNA Library Prep Kit for Illumina^®^ (NEB, E7530L) according to the manufacturer’s instructions. The cDNA libraries were subjected to the NovaSeq 6000 system (Illumina), according to commercially available protocols. The changed RNAs were validated by quantitative PCR using the primers listed in Table S2.

### 2.22. RNA-Seq Analysis

The quality control of raw sequencing data was conducted using fastp [42] The clean reads from RNA-seq were aligned to the human reference genome sequence, GRCH38.p13, using the HISAT2 program (v2.2.3) [43] The gene expression level was determined by the featurecounts function of subread software with the genome annotation file from GENCODE (v36) [44,45,46].

### 2.23. Statistical Analysis

Data were showed as the mean values with the standard error of the mean. Statistical differences were determined using Student’s *t*-test or one-way ANOVA. *p* < 0.05 was considered to indicate statistical significance.

## 3. Results

### 3.1. The Highly Expressed KAT2A and E2F1 Could Promote Cancer Progress in Pan-Cancer

**Figure 1:**
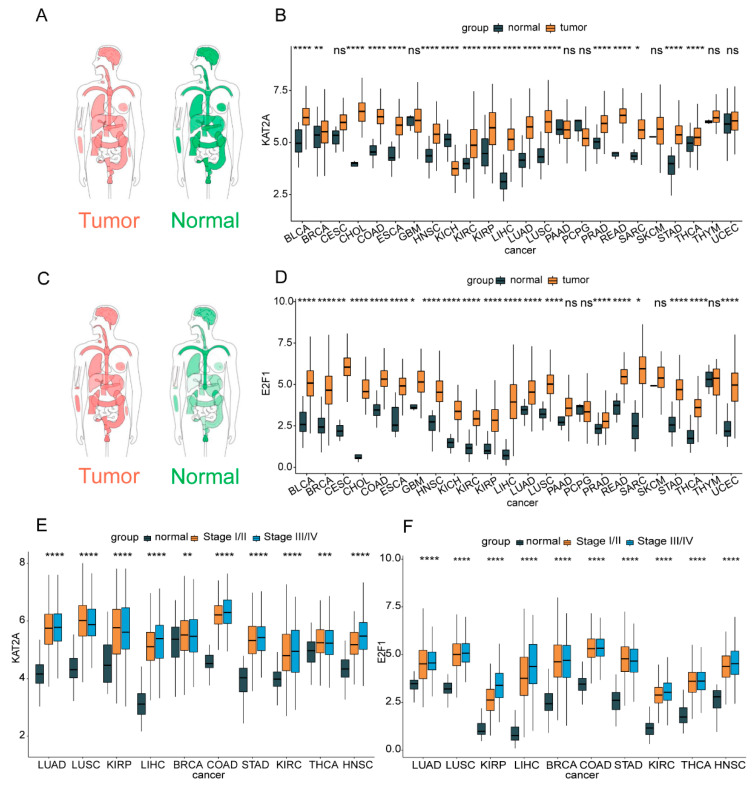
The expression levels of *KAT2A* and *E2F1* in pan-cancer tissues and cells. The expression profiles of *KAT2A* (**A**,**C**), *E2F1* in a normal person and patients. The darker the color, the higher the gene expression. The expression levels of *KAT2A* (**B**) and *E2F1* (**D**) in TCGA pan-cancer tissue and normal tissue samples. The expression levels of *KAT2A* (**E**) and *E2F1* (**F**) in pathological stages. The statistical significance was analyzed by Student’s *t*-test (two-tailed) analysis. ACC, adrenocortical carcinoma; BLCA, bladder urothelial carcinoma; BRCA, breast invasive carcinoma; CESC, cervical squamous cell carcinoma and endocervical adenocarcinoma; CHOL, cholangiocarcinoma; COAD, colon adenocarcinoma; DLBC, lymphoid neoplasm diffuse large B-cell lymphoma; ESCA, esophageal carcinoma; GBM, glioblastoma multiforme; HNSC, head and neck squamous cell carcinoma; KICH, kidney chromophobe; KIRC, kidney renal clear cell carcinoma; KIRP, kidney renal papillary cell carcinoma; LAML, acute myeloid leukemia; LGG, brain lower grade glioma; LIHC, liver hepatocellular carcinoma; LUAD, lung adenocarcinoma; LUSC, lung squamous cell carcinoma; MESO, mesothelioma; OV, ovarian serous cystadenocarcinoma; PAAD, pancreatic adenocarcinoma; PCPG, pheochromocytoma and paraganglioma; PRAD, prostate adenocarcinoma; READ, rectum adenocarcinoma; SARC, sarcoma; SKCM, skin cutaneous melanoma; STAD, stomach adenocarcinoma; TGCT, testicular germ cell cancers; THCA, thyroid carcinoma; THYM, thymoma; UCEC, uterine corpus endometrial carcinoma; UCS, uterine carcinosarcoma; UVM, uveal melanoma. *: *p* < 0.05; **: *p* < 0.01; ***: *p* < 0.001; ****: *p* < 0.0001; ns: no significance.

From the GEPIA online analysis, we found that compared with the expression level distributed in a normal person, *KAT2A* was slightly higher in cancer patients, and *E2F1* was obviously higher expressed in cancer patients (Figure 1A,C) [47,48]. As a result of the TCGA pan-cancer transcriptome data, the expression levels of *KAT2A* and *E2F1* were significantly up-regulated in 16 and 20 kinds of tumor tissues compared with normal tissues, respectively. Both the expression levels of *KAT2A* and *E2F1* were significantly up-regulated in the same 16 cancers, and *E2F1* was also higher expressed in CESC, GBM, KICH, and UCEC cancers (Figure 1B,D). In addition, the expression levels of *KAT2A* and *E2F1* in pathological stages were remarkably higher expressed in stage III and IV in 10 cancers (Figure 1E,F). These findings suggested that *KAT2A* and *E2F1* may play an important role in tumor development.

**Figure 2:**
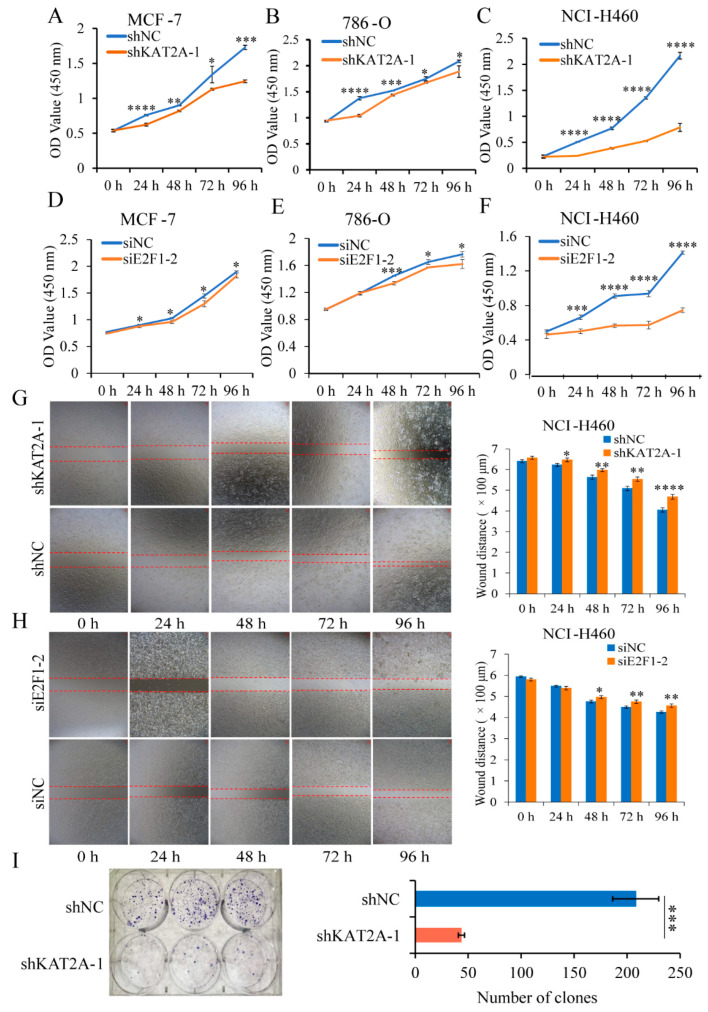
Interfering with *KAT2A* and *E2F1* could significantly inhibit the proliferation and migration of cancer cells. (**A**–**C**) After transfection of shNC and shKAT2A-1, the cell proliferation ability of MCF-7 (**A**), 786-O (**B**), and NCI-H460 (**C**) were observed using CCK-8. (**D**–**F**) After transfecting siNC and siE2F1-2, the cell proliferation ability of MCF-7 (**D**), 786-O (**E**), and NCI-H460 (**F**) was detected by CCK-8 assay. Data are representative from at least six independent experiments. The NCI-H460 cells’ migration ability after interference with *KAT2A* (**G**) and *E2F1* (**H**), and their negative control (NC), respectively. Data are representative from three independent experiments. (**I**) Clone formation of NCI-H460 cells with a stable knockdown of KAT2A-1 and NC. Data are representative from three independent experiments. The statistical significance was analyzed by Student’s *t*-test (one-tailed) analysis. *: *p* < 0.05; **: *p* < 0.01; ***: *p* < 0.001; ****: *p* < 0.0001.

From the results of CCK-8 and wound-healing assays, the proliferation of MCF-7, 786-O, and NCI-H460 cells (Figure 2A–F), and the cell migration ability of NCI-H460 cells (Figure 2G,H) were suppressed with the knockdown of either *KAT2A* or *E2F1*, compared with the control group. Moreover, the knockdown of *KAT2A* significantly inhibited the colony-forming ability of NCI-H460 cells (Figure 2I).

### 3.2. UBE2C May Be a Downstream Gene That Is Co-Regulated by KAT2A/E2F1 in Pan-Cancer

**Figure 3:**
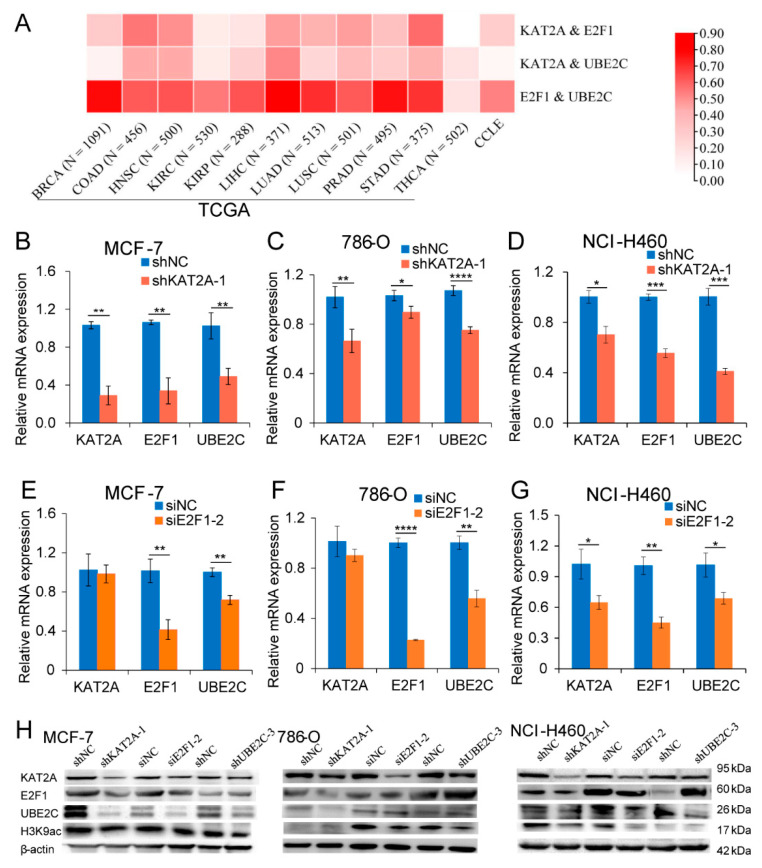
Both *KAT2A* and *E2F1* could regulate the expression of *UBE2C* in different cancers. (**A**) The correlation coefficient between *KAT2A*, *E2F1*, and *UBE2C* in different cancers. (**B**–**D**) The expression levels of *KAT2A*, *E2F1*, and *UBE2C* were detected by qRT-PCR after interference with *KAT2A* and NC in MCF-7 (**B**), 786-O (**C**), and NCI-H460 (**D**) cells. (**E**–**G**) qRT-PCR detected the expression levels of *KAT2A*, *E2F1*, and *UBE2C* after interference with *E2F1* and NC in MCF-7 (**E**), 786-O (**F**), and NCI-H460 (**G**) cells. Data are representative from at least three independent experiments. N = 3. The statistical significance was analyzed by Student’s *t*-test (one-tailed) analysis. (**H**) After interfering with *KAT2A*, *E2F1*, and *UBE2C* and their corresponding NC, the expression levels of their encoded proteins in MCF-7, 786-O, and NCI-H460 cells were detected using Western blot, respectively. N = 1. *: *p* < 0.05; **: *p* < 0.01; ***: *p* < 0.001; ****: *p* < 0.0001.

In order to explore the potential target genes co-regulated by *KAT2A* and *E2F1*, the differentially expressed genes (DEGs) of *KAT2A* and *E2F1* were screened according to the conditions. A total of 9 to 1202 overlapping DEGs were obtained from different cancer types (Figure S1A–K). Among 11 cancers, there were 222 genes that appeared in more than five cancer types at the same time (Figure S1L), including *UBE2C*, which appeared in six types of cancer (Table S3). The GO analysis of the overlapping 222 DEGs revealed that the biological pathways (BP) were related to DNA replication, nuclear division, the regulation of cell cycle phase transition and cell cycle checkpoint, and so on (Figure S2A); the enriched cell components (CC) included chromosomal region, chromosome, centromeric region, spindle, and kinetochore (Figure S2B); and the molecular function (MF) was involved in the action of catalytic activity, acting on DNA, deoxyribonuclease activity, and so on (Figure S2C). The KEGG pathway analysis indicated that these genes were significantly enriched in the cell cycle, DNA replication, homologous recombination, base excision repair and nucleotide excision repair, and so on (Figure S2D). From the result of correlation analysis in tumor tissue samples across 11 cancer types and cell line samples in CCLE, we found that the correlation coefficient between the two in *KAT2A*, *E2F1*, and *UBE2C* was 0.037–0.82 (Figure 3A). Considering the analysis results of the transcriptional data of various cancers in the TCGA database (Figure S1), it is suggested that *UBE2C* may be a downstream gene that is co-regulated by *KAT2A* and *E2F1*. Correspondingly, we found that both the mRNA and the protein levels of UBE2C were significantly suppressed after the knockdown of *KAT2A* or *E2F1* in MCF-7, 786-O, and NCI-H460, respectively (Figure 3B–H). Moreover, the mRNA/protein changes of *E2F1* were significantly inhibited after knocking down *KAT2A* (Figure 3B–D). Furthermore, the knockdown of *UBE2C* caused an extreme decrease of the *KAT2A* protein level (Figure 3H).

**Figure 4:**
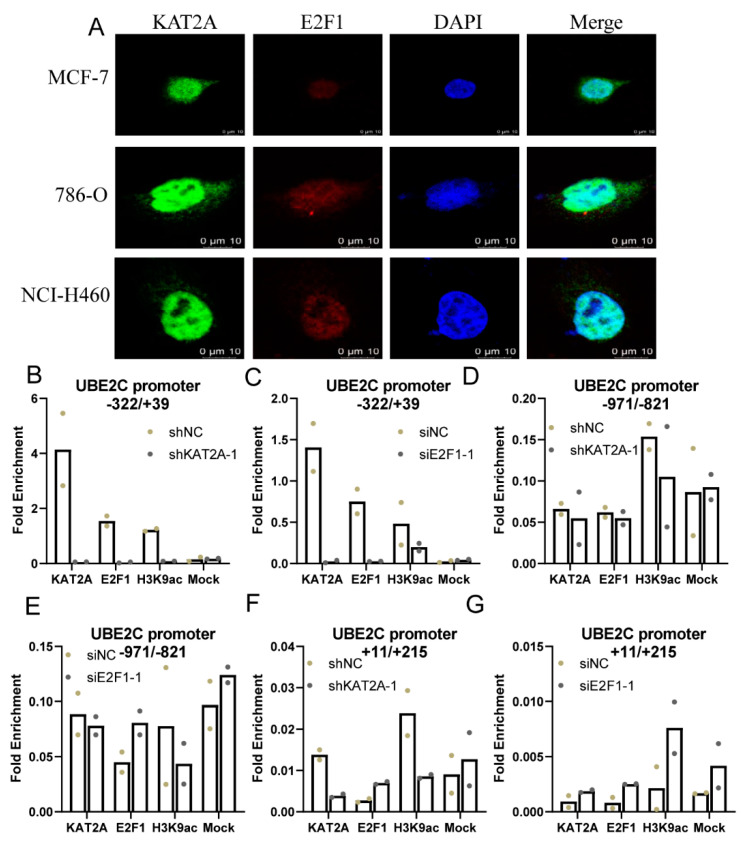
*KAT2A* and *E2F1* bind to the *UBE2C* promoter in different cancer cells. (**A**) Cellular immunofluorescence showed the nuclear localization of *KAT2A* and *E2F1* in MCF-7, 786-O, and NCI-H460 cells. (**B**–**G**) After interference with *KAT2A* and *E2F1* and their corresponding NC, respectively, the enrichment of *KAT2A*, *E2F1*, and H3K9ac on the *UBE2C* promoter. Data are representative from two independent experiments. N = 2. The bar graph represents mean values, and the dot plots of individual data points are overlaid on bar graphs.

Additionally, the results of cellular immunofluorescence showed the nuclear localization of *KAT2A* and *E2F1* in MCF-7, 786-O, and NCI-H460 cells (Figure 4A). Moreover, ChIP-qPCR showed that *KAT2A* and *E2F1* can bind to the −322 to +39 region of the *UBE2C* promoter (with more than one-fold enrichment), not −1081 to −819, or +11 to +215 regions (with less than one-fold enrichment) (Figure 4B–G). Additionally, the dual luciferase reporter gene experiment also confirmed that *E2F1* could bind to the −273 to −266 region of the *UBE2C* promoter (Figure S3A–F). Moreover, the ChIP-qPCR assay demonstrated that the *KAT2A* bound to the promoter region of *UBE2C*, and increased the H3K9 acetylation level in this promoter region, which suggested that *KAT2A* and *E2F1* may cooperate to regulate *UBE2C* gene transactivation via histone modification. Moreover, the Co-IP assays showed that *KAT2A*, *E2F1*, and H3K9ac could bind to each other (Figure S3G). These results demonstrated that *KAT2A* may promote the expression of *UBE2C* through combining with *E2F1* to the *E2F1* binding site on *UBE2C* promoter −322/+39 region to increase the acetylation level of H3K9, and consequently stimulated cancer cell proliferation and migration.

### 3.3. UBE2C Highly Expresses in Pan-Cancer

**Figure 5:**
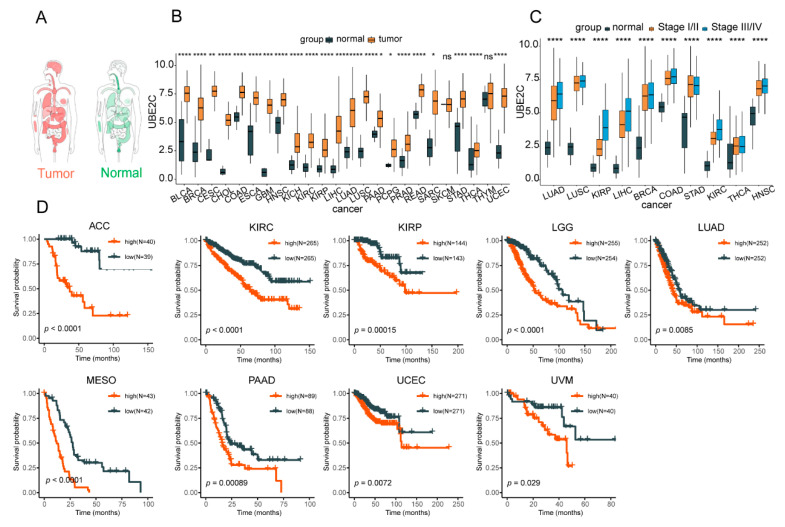
*UBE2C* significantly highly expresses in pan-cancer tissues and cells. (**A**) The expression profile of *UBE2C* in a normal person and patients. The darker the color, the higher the gene expression. (**B**) The expression level of *UBE2C* in TCGA pan-cancer tissues and their corresponding normal tissues. (**C**) The expression level of *UBE2C* in pathological stages of different cancer types. The statistical significance was analyzed by Student’s *t*-test (two-tailed) analysis. (**D**) The survival analysis of *UBE2C* in different cancer types. Kaplan–Meier survival analysis and the log-rank test were employed to compare OS between the tumor and normal cohorts. *: *p* < 0.05; **: *p* < 0.01; ***: *p* < 0.001; ****: *p* < 0.0001; ns: no significance.

The results of the GEPIA online analysis and TCGA database showed that the expression of *UBE2C* was higher in many types of tumors than normal tissues, especially in brain, lung, and breast (Figure 5A,B). Additionally, the result of the expression levels of *UBE2C* in pathological stages showed that it was significantly highly expressed in stage III and IV in 10 types of cancer (Figure 5C). In addition, based on our previous study [39], the analysis of tumor tissue single-cell transcriptome data showed that the expression of *UBE2C* has a significant up-regulation trend in cancer cell clusters (CS), such as CS4 of CRC, CS2 of LC, CS4 of OV, CS3 of PDAC, and CS5 of SCC (Figure S4). Moreover, the survival analyses revealed that the high expression of *UBE2C* was significantly associated with a poor prognosis in patients of nine cancer types (Figure 5D). The above results suggested that *UBE2C* was up-regulated in a variety of cancer tissues/cells, and it may play a pivotal role in the development of pan-cancer.

### 3.4. Knockdown of UBE2C Significantly Inhibits Cancer Cell Proliferation and Migration, and Promotes Cell Apoptosis

**Figure 6:**
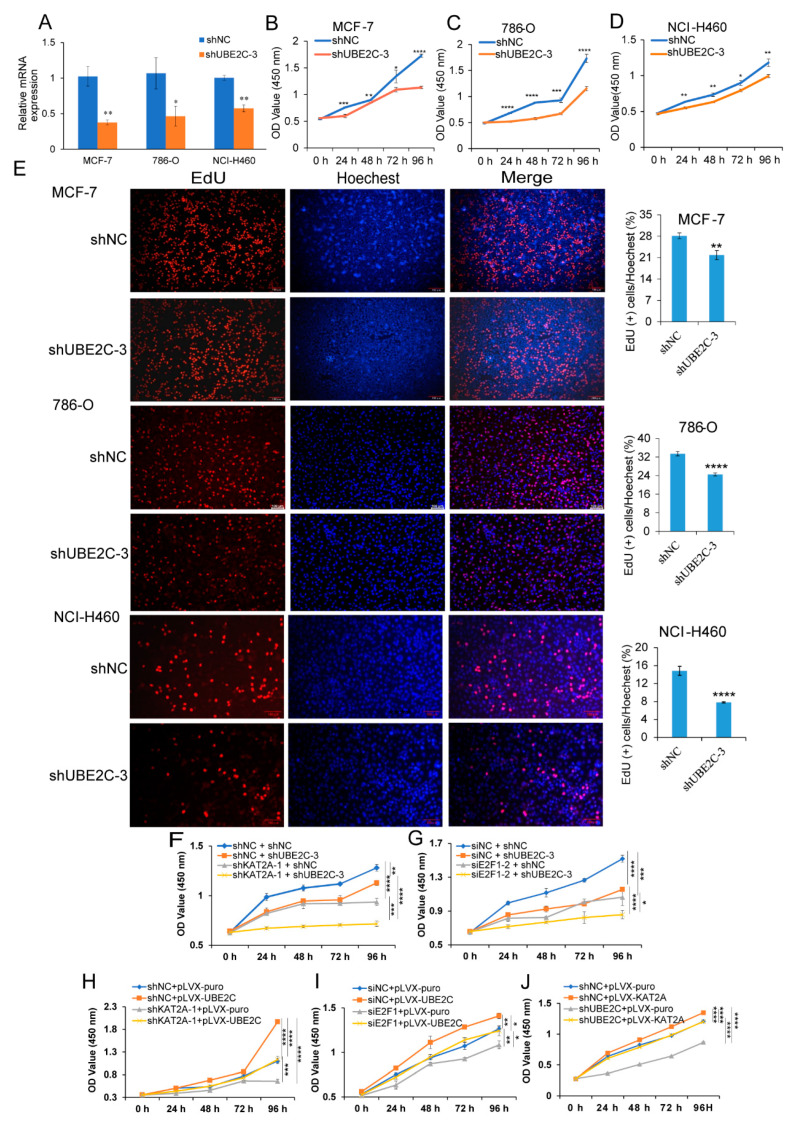
Interference with *UBE2C* significantly inhibits cancer cell proliferation. (**A**) The expression level of *UBE2C* was detected by qRT-PCR after interference with *UBE2C* and NC in MCF-7, 786-O, and NCI-H460 cells. Data are representative from four independent experiments. CCK-8 results of MCF-7 (**B**), 786-O (**C**), and NCI-H460 (**D**) cells interfered with *UBE2C* and NC at different time points. Data are representative from at least six independent experiments. (**E**) EdU results of MCF-7, 786-O, and NCI-H460 cells interfered with *UBE2C* and NC at 48 h. EdU marks the proliferating cells, Hoechest represents the nucleus, and Merge represents an overlay of EdU and Hoechest. 10× magnification. Data are representative from three independent experiments. CCK-8 results of separately or jointly interfered with *KAT2A* and *UBE2C* (**F**), or *E2F1* and *UBE2C* (**G**), and corresponding control at different time points. CCK-8 results of interference with *KAT2A* (**H**) or *E2F1* (**I**), but overexpressed *UBE2C* and their corresponding control groups at different time points. (**J**) CCK-8 results of interference with *UBE2C*, but overexpressed *KAT2A* and their corresponding control groups at different time points. Data are representative from eight independent experiments. The statistical significance was analyzed by Student’s *t*-test (one-tailed) analysis. *: *p* < 0.05; **: *p* < 0.01; ***: *p* < 0.001; ****: *p* < 0.0001.

To investigate the function of *UBE2C* in MCF-7, 786-O, and NCI-H460 cells, we transfected the cell lines with UBE2C shRNA (shUBE2C), and the qRT-PCR results showed that the expression of *UBE2C* in shUBE2C-treated group was significantly lower than that in the negative control group (shNC) (Figure 6A). Additionally, the results from the CCK-8 assay showed that the proliferation of different cancer cell lines was markedly suppressed with knocking down *UBE2C* in MCF-7, 786-O, and NCI-H460 cells compared with the control group (Figure 6B–D). As a key downstream gene of *KAT2A* and *E2F1*, we also found that *UBE2C* significantly inhibited the proliferation of the liver cancer cell line, HepG2, and pancreatic cancer cell line, BxPC3, through a CCK-8 assay (Figure S5), which suggested that *UBE2C* plays a critical role in pan-cancer. The EdU experiment suggested that the number of proliferating cells was significantly reduced after the knockdown of *UBE2C* at 48 h (Figure 6E). In addition, we found that the cell proliferation ability was significantly inhibited after co-interfering with *KAT2A* and *UBE2C*, *E2F1*, and *UBE2C*, compared to that of only knocking down *KAT2A* or *E2F1* in NCI-H460 cells (Figure 6F,G). Moreover, compared with the control group (pLVX-puro), the overexpression of *UBE2C* (pLVX-*UBE2C*) significantly promoted cell proliferation, whereas knocking down *KAT2A* and *E2F1* caused an inhibition on cell proliferation, which can be restored by the overexpression of *UBE2C* (Figure 6H,I). Moreover, the overexpression of *KAT2A* (pLVX-KAT2A) also significantly promoted cell proliferation, whereas knocking down *UBE2C* caused an inhibition on cell proliferation, which can be restored by the overexpression of *KAT2A* (Figure 6J). These results not only suggested that the downregulation of *UBE2C* could significantly inhibit the proliferation of tumor cells, but also confirmed that *KAT2A* and *E2F1* could indeed affect the proliferation of tumor cells through regulating the expression of *UBE2C*.

Next, we examined the effects of *UBE2C* on cell metastasis and apoptosis. The results of the wound-healing assay (Figure S6A–C) and transwell experiment (Figure S7A–C) showed that the downregulation of *UBE2C* remarkably suppressed the cell migration ability in MCF-7, 786-O, and NCI-H460 cells. Moreover, the number of apoptotic cells in MCF-7, 786-O, and NCI-H460 cells (the total number of cells in Q2 and Q3 in the figure) was significantly higher after 48 h of knockdown of *UBE2C* than that of the control groups (Figure S8A–C).

### 3.5. UBE2C Promotes the Development of Pan-Cancer through Influencing the Cell Cycle

**Figure 7:**
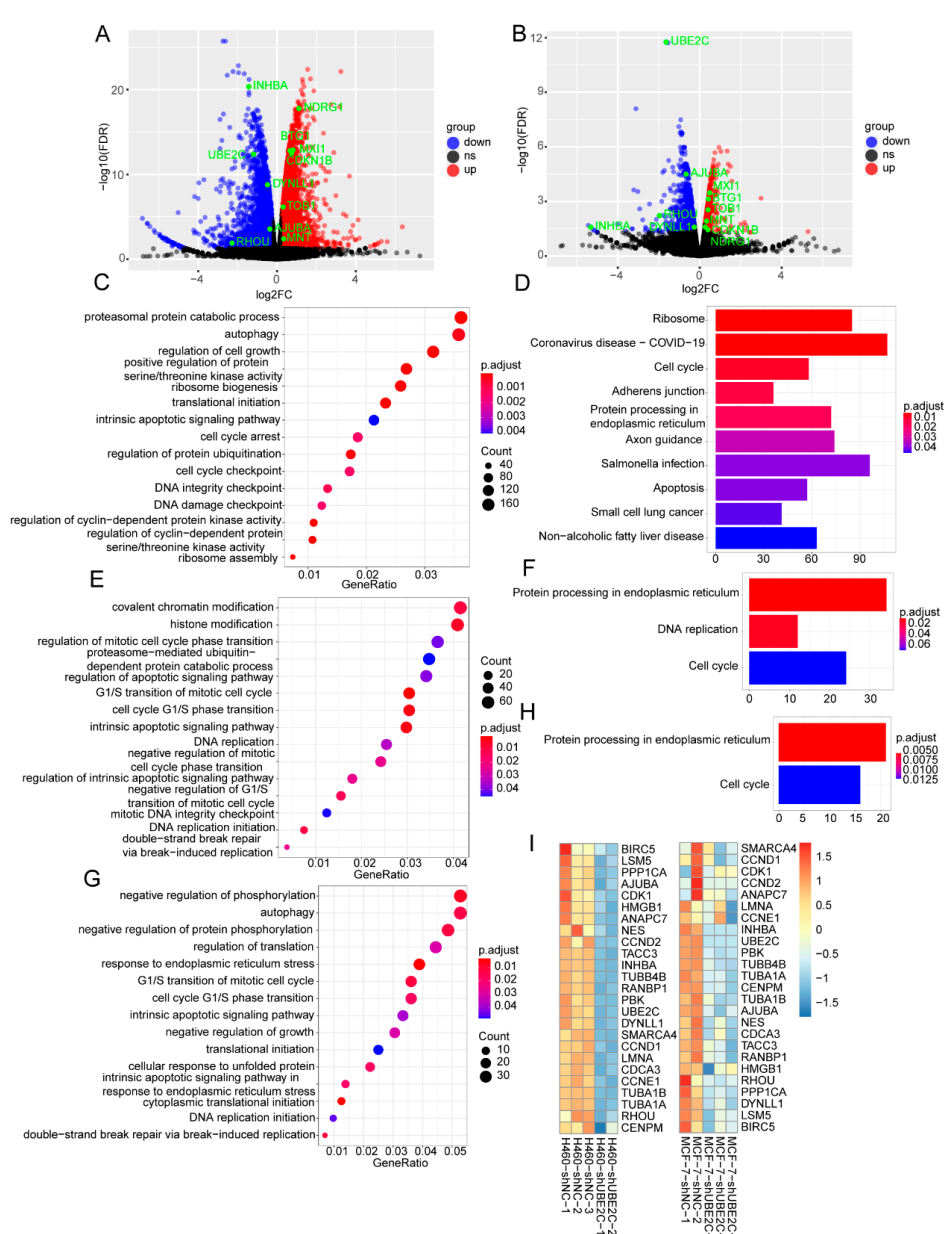
Functional enrichment analysis of RNA-Seq DEGs. (**A**,**B**) The volcano maps of NCI-H460 (left panel) and MCF-7 (right panel) DEGs with threshold of |FC| > 1.2 and FDR < 0.05. ns: no significance. (**C**,**D**) The GO_BP and KEGG pathways of NCI-H460 DEGs, respectively. (**E**,**F**) The GO_BP and KEGG pathways of MCF-7 DEGs, respectively. (**G**,**H**) The GO_BP and KEGG pathways of the overlap DEGs between NCI-H460 and MCF-7 cells, respectively. (**I**) The cell-cycle-related genes from both TCGA and RNA-seq were clustered using hierarchical clustering based on Euclidean distance. The color represents the TPM of different genes from shUBE2C and shNC groups of NCI-H460 and MCF-7 cancer cell lines.

To better understand the molecular signatures after the knockdown of *UBE2C* in NCI-H460 and MCF-7 cells, we performed RNA-seq analysis and analyzed the DEGs. As a result, a total of 5539 and 1756 DEGs in NCI-H460 and MCF-7 cancer cells was screened with |FC| > 1.2 and FDR < 0.05, respectively (Figure 7A,B). As expected, the significantly enriched pathways of GO_BP of NCI-H460 were translational initiation, regulation of cell growth, cell cycle arrest, cell cycle checkpoint, and so on (*p*.adjust < 0.05) (Figure 7C). The KEGG pathways of NCI-H460 were the included ribosome, cell cycle, adherens junction, protein processing in endoplasmic reticulum, and apoptosis (Figure 7D). Additionally, the GO analysis showed that the DEGs of MCF-7 were highly associated with cell cycle G1/S phase transition, the intrinsic apoptotic signaling pathway, histone modification, and DNA replication initiation (Figure 7E). The KEGG analysis showed that the DEGs of MCF-7 were significantly related to protein processing in the endoplasmic reticulum, DNA replication, and the cell cycle (Figure 7F). These results suggested that *UBE2C* may promote the development of pan-cancer through influencing the cell cycle.

Notably, there were 772 overlap DEGs in the two cell lines, including Cyclin Dependent Kinase Inhibitor 1B (*CDKN1B*), Inhibin Subunit β A (*INHBA*), Ras Homolog Family Member U (*RHOU*), BTG Anti-Proliferation Factor 1 (*BTG1*), Transducer Of ERBB2, 1 (*TOB1*), N-Myc Downstream Regulated 1 (*NDRG1*), MAX Network Transcriptional Repressor (*MNT*), MAX Interactor 1, Dimerization Protein (*MXI1*), and Ajuba LIM Protein (*AJUBA*), which have been reported to be involved in the cell cycle and proliferation [49,50,51,52,53,54,55,56]. Intriguingly, the GO analysis showed that the overlap genes were highly associated with the G1/S transition of the mitotic cell cycle, intrinsic apoptotic signaling pathway, and negative regulation of growth and DNA replication initiation (Figure 7G), and the KEGG analysis showed that they were significantly related to cell cycle and protein processing in the endoplasmic reticulum (Figure 7H). Moreover, the heat map showed that the DEGs in two cancer cell lines with interfering *UBE2C* was involved in the cell cycle (Figure 7I), suggesting that UBE2C participating in the cell cycle may be a common regulatory mechanism of pan-cancer development. Therefore, we performed cell cycle experiments and found that the cell cycle was significantly blocked in the G1 phase after the knockdown of *UBE2C*, suggesting that *UBE2C* may affect the cell cycle by regulating the G1/S phase transition (Figure S9).

To further validate how *UBE2C* affects the progress of cancer development through the cell cycle, the DEGs were obtained based on the 30% of high and low expression levels of *UBE2C* in 11 types of cancer tissues. A total of 1514 DEGs, which appeared in five types of cancer, were screened. A total of 596 DEGs was obtained from 1514 DEGs overlapped with the DEGs from the GSE173127 dataset. The GO analysis of these 596 DEGs revealed that they were mainly involved in organelle fission, DNA replication, and the cell cycle checkpoint (Figure S10A–C), and the KEGG analysis showed that they were also involved in the cell cycle, DNA replication, and p53 signaling pathway (Figure S10D). The PPI analysis results of the above 596 DEGs showed that UBE2C can form an interaction network with Tumor-Transforming Protein 1 (PTTG1), Polo Like Kinase 1 (PLK1), Cyclin Dependent Kinase 1 (CDK1), Cell Division Cycle 20 (CDC20), cyclin B2 (CCNB2), cyclin B1 (CCNB1), cyclin A2 (CCNA2), and Breast and Ovarian Cancer Susceptibility Protein 1 (BRCA1) (Figure S10E). Genes regulated by *UBE2C* can also form an interaction network with TP53 (Figure S10F). Furthermore, hub genes contained kinesin family members 11 and 20A (KIF11, KIF20A), BUB1 mitotic checkpoint serine/threonine kinase (BUB1), Aurora Kinase B (AURKB), CCNA2, CCNB2, CDC20, CDK1, topoisomerase (DNA) II α (TOP2A), and DLG associated protein 5 (DLGAP5) (Figure S10G).

## 4. Discussion

Multiple studies have revealed that *KAT2A* was highly expressed in a variety of cancers compared with adjacent tissues, such as liver cancer [57], colon adenocarcinoma tissues [58], and non-small cell lung cancer tissues [16]. The downregulation of *KAT2A* can significantly reduce the proliferation and migration of cancer cells and the growth of xenograft tumors [57,59]. In this study, we found that *KAT2A* was generally highly expressed in seven cancer tissues compared with normal tissues, including BLCA, CHOL, ESCA, and HNSC, KIRP, STAD, and THCA. As a transcription factor, high *E2F1* levels were commonly associated with aggressive cancer and poor patient prognosis for multiple cancer types. *E2F1* transcription factor was a key regulator of genes required for cell cycle progression, cell proliferation, and differentiation [21,60], and played a key regulatory role in the invasion–metastasis cascade of certain cancer types [61,62]. Previous studies have revealed that *E2F1* can induce cell metastasis by inducing chemoresistance, angiogenesis, secondary site extravasation, and EMT [63,64,65,66,67,68,69] This study found that *E2F1* was significantly highly expressed in 20 cancers, and it was significantly related to the poor prognosis in patients of 9 different cancers.

By screening the transcriptome data of 11 cancer tissues of TCGA, it was exposed that the gene set potentially co-regulated by *KAT2A* and *E2F1* was significantly enriched in the cell cycle, and participated in DNA replication, base excision repair, and nucleotide excision repair. It was suggested that the regulatory network of *KAT2A* and *E2F1* involved in the cancer process was very complex, and it could regulate the expression level of genes involved in the cancer development process.

Previous studies have shown that *KAT2A* could increase the chromatin accessibility of *E2F1*, DNA Damage Inducible Transcript 3 (DDIT3), and other transcription factors, and form protein complexes with them, and then be recruited to the promoter regions of related genes, consequently enhancing their expression through increasing the acetylation level of H3K9 on these gene-promoting regions and regulating the development of cancer. In this study, the ChIP-qPCR and Co-IP results have demonstrated that *KAT2A* may interact with *E2F1* and form a complex to bind the *UBE2C* promoter in MCF-7, 786-O, and NCI-H460 cells. The complex could increase the level of H3K9ac, thereby promoting the expression of *UBE2C*. Moreover, we uncovered that the *E2F1* binding site region (−322/+39) of the *UBE2C* promoter was consistent with the results of a previous study [70].

This study explored the regulatory relationship between *KAT2A*/*E2F1* and *UBE2C*, and found that *KAT2A* can regulate the expression of *UBE2C* through interacting with *E2F1*. KAT2A can also affect the expression of *E2F1*, which is in agreement with previous studies [16,71]. *E2F1* can also regulate the expression of *KAT2A*, but whether it affects the expression of *KAT2A* through the *E2F1* binding site on the *KAT2A* promoter or other mechanisms still needs further in-depth research. Interestingly, the downregulation of *UBE2C* can inhibit *KAT2A* protein expression levels, indicating that *UBE2C* can regulate the expression of *KAT2A* and form feedback regulation. Previous studies have reported that the SCF-Cyclin F ubiquitin ligase complex participates in the ubiquitination and degradation of *E2F1* [72], but whether its degradation required the participation of *UBE2C* has not been reported. If it was required for the involvement of *UBE2C*, it can be explained that the *E2F1* protein was significantly upregulated in 786-O and NCI-H460 after the knockdown of *UBE2C* for the reason that the degradation of the *E2F1* protein was reduced after the knockdown of *UBE2C*. However, what role these up-regulated *E2F1* proteins may play need further research.

Given the clinical and functional significance of *KAT2A*/*E2F1*/*UBE2C* in pan-cancer, we concluded that *KAT2A*/*E2F1*/*UBE2C* and its associated pathway were crucial for cancer carcinogenesis, and targeting this pathway may be pivotal in the prevention or treatment of pan-cancer.

## Data Availability

The data analyzed during the present study are available from the corresponding author upon reasonable request.

## Supplementary Materials

The following supporting information can be downloaded at: https://www.mdpi.com/article/10.3390/genes13101817/s1, Figure S1. Potential target genes co-regulated by *KAT2A* and *E2F1*; Figure S2. GO and KEGG enrichments of *KAT2A* and *E2F1* target genes; Figure S3. *KAT2A* and *E2F1* regulate the transcription of *UBE2C* through *E2F1* binding site; Figure S4. The expression level of *UBE2C* in different cell types in cancer tissues; Figure S5. Interference with *UBE2C* significantly inhibits cancer cell proliferation; Figure S6. Interference with *UBE2C* significantly inhibited cancer cell migration; Figure S7. Interfering *UBE2C* could remarkably repress cancer cell invasion; Figure S8. Interference with *UBE2C* significantly promoted cancer cell apoptosis; Figure S9. Interference with *UBE2C* significantly induced cell cycle arrest; Figure S10. Top 15 of GO terms, top 12 of KEGG signaling pathways and PPI network of 596 DEGs that are regulated by *UBE2C*; Table S1. Summary of cancer and normal samples analyzed in this study; Table S2. Primers and interfering sequence for target genes used in this study; Table S3. A total of 222 DEGs regulated by *KAT2A* and *E2F1* appeared in more than five cancer types among 11 different types of cancer at the same time.
