# Supplementary material for: KAT2A/E2F1 Promotes Cell Proliferation and Migration via Upregulating the Expression of UBE2C in Pan-Cancer
Source: Genes (Basel). 2022 Oct 8;13(10):1817. doi: 10.3390/genes13101817 (PMC9602169; doi:10.3390/genes13101817)
Supplement: Supplementary file 1 [file genes-13-01817-s001.zip › Supplementary Material.pdf]

Supplementary Material

The Supplementary Material for this article can be found online at:

Supplementary Figures

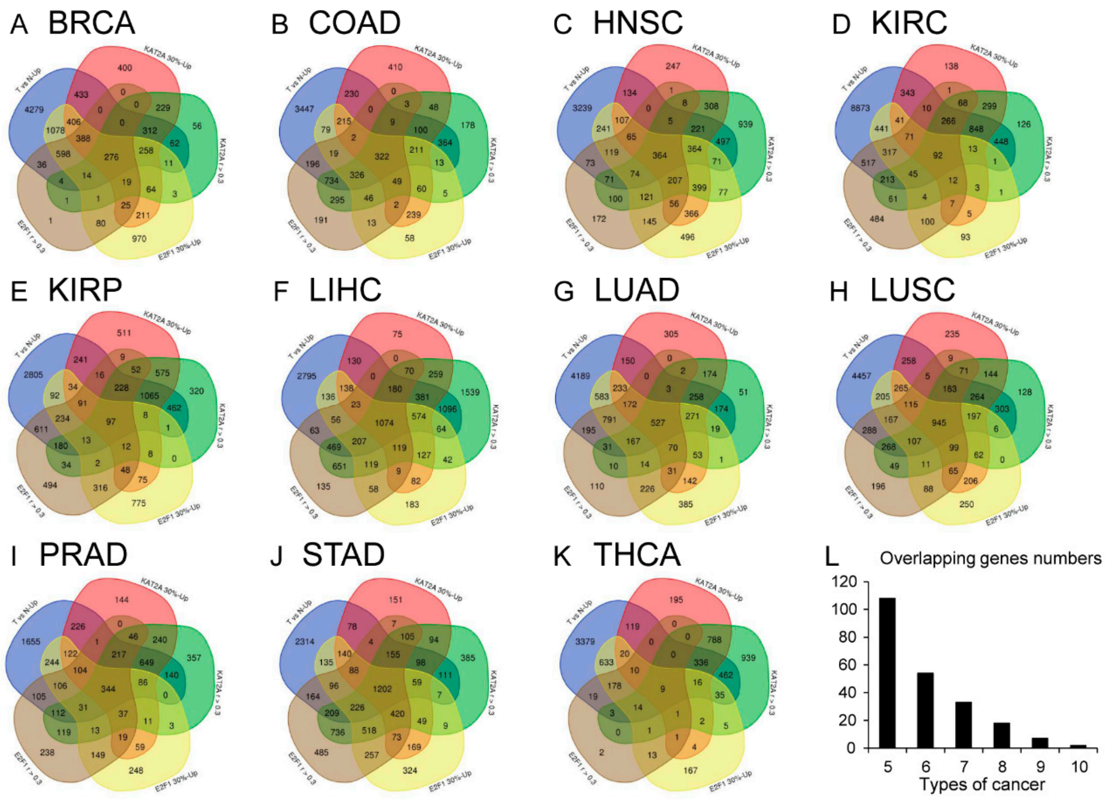

Figure S1 Potential target genes co-regulated by *KAT2A* and *E2F1*. (A) BRCA; (B) COAD; (C) HNSC; (D) KIRC; (E) KIRP; (F) LIHC; (G) LUAD; (H) LUSC; (I) PRAD; (J) STAD; (K) THCA; (L) the number of DEGs that appear in more than 5 cancers.

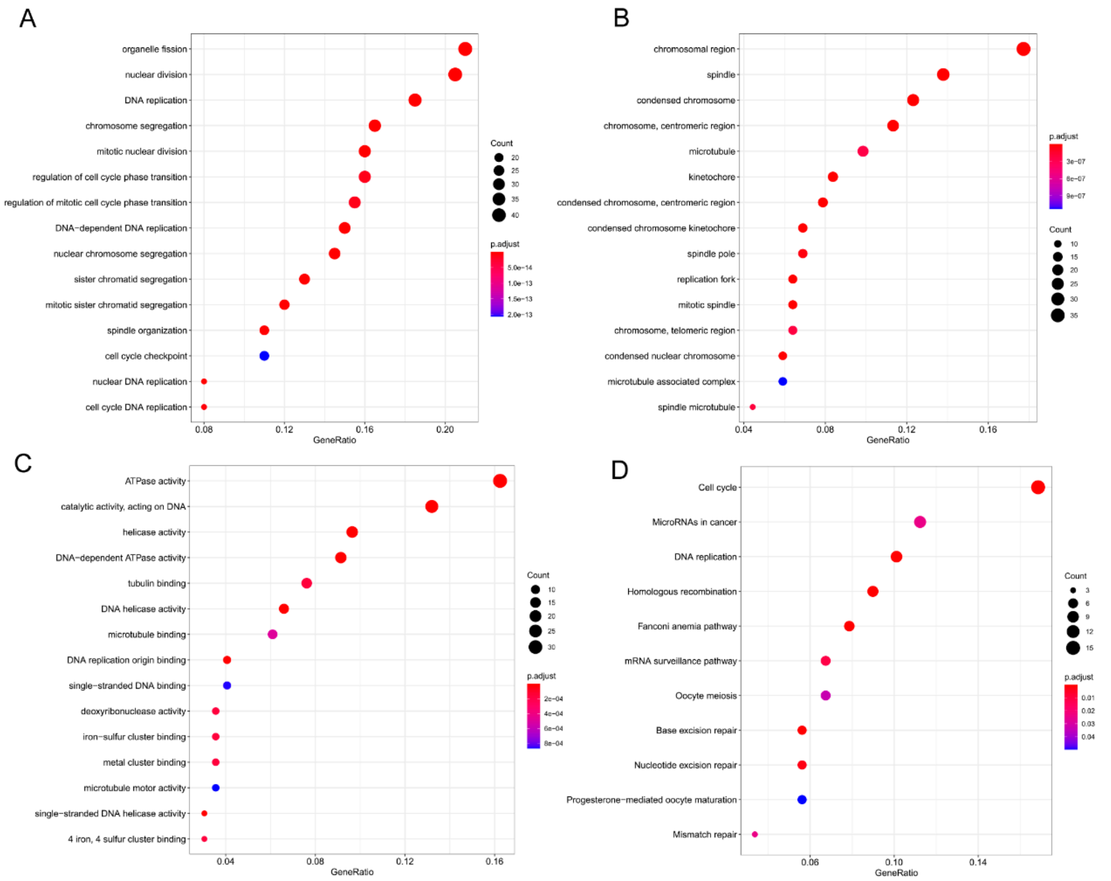

Figure S2 GO and KEGG enrichments of *KAT2A* and *E2F1* target genes. GO and KEGG enrichments of *KAT2A* and *E2F1* target genes Significant enrichment pathways of (A) GO_CC, (B) GO_BP, (C) GO_MF and (D) KEGG.

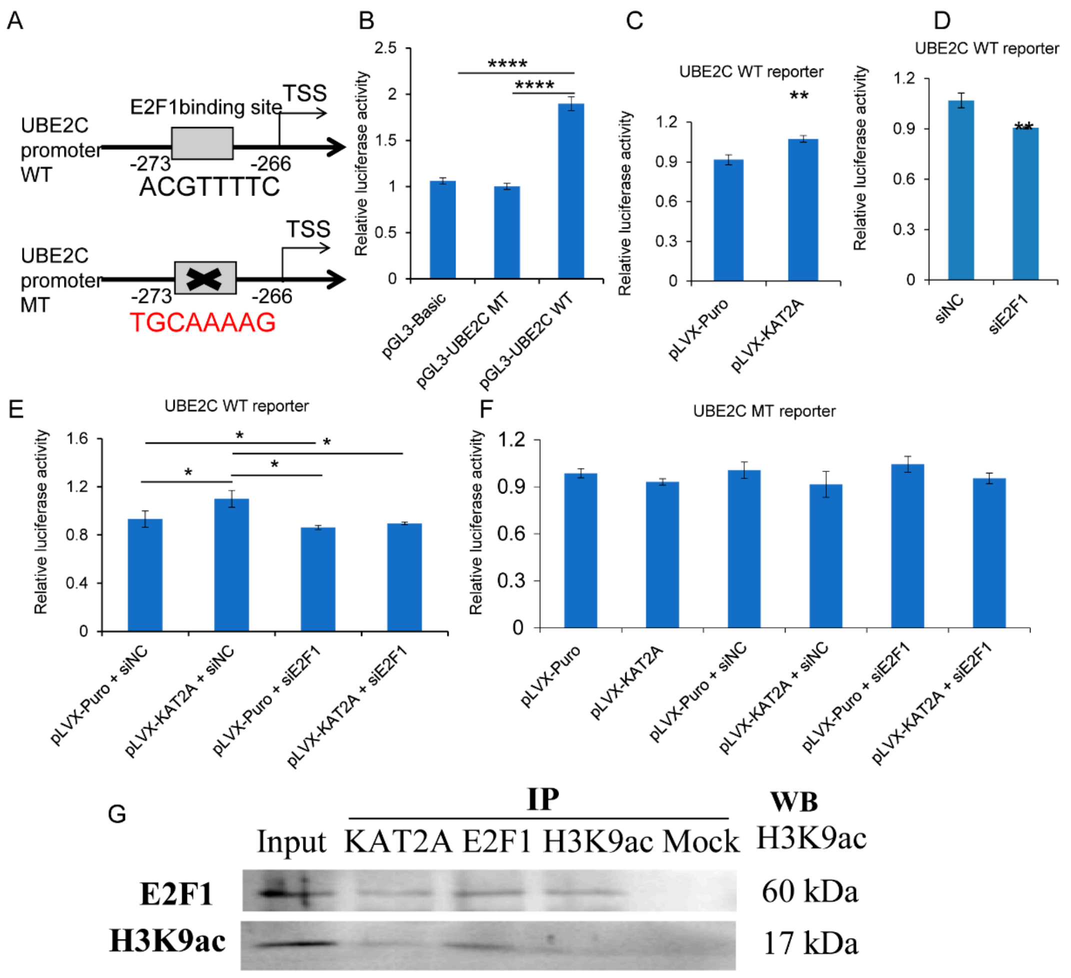

Figure S3 *KAT2A* and *E2F1* regulate the transcription of *UBE2C* through *E2F1* binding site. (A) The schematic diagram of *UBE2C* promoter with *E2F1* binding site in wild type and mutant. (B) The transcriptional activity of wild-type and mutant *UBE2C* promoters. The transcriptional activity of wild-type *UBE2C* promoter with overexpression of *KAT2A* (C) and interference with *E2F1* (D). The transcriptional activity of wild-type (E) and mutant (F) *UBE2C* promoters with overexpression of *KAT2A* alone or at the same time with interference of *E2F1*. Data are representative from three independent experiments. The statistical significance was analyzed by Student's t-test (one-tailed) analysis. (G) Co-IP result among KAT2A, E2F1 and H3K9ac. *: $p< 0.05$; **: $p < 0.01$; ***: $p < 0.001$; ****: $p < 0.0001$.

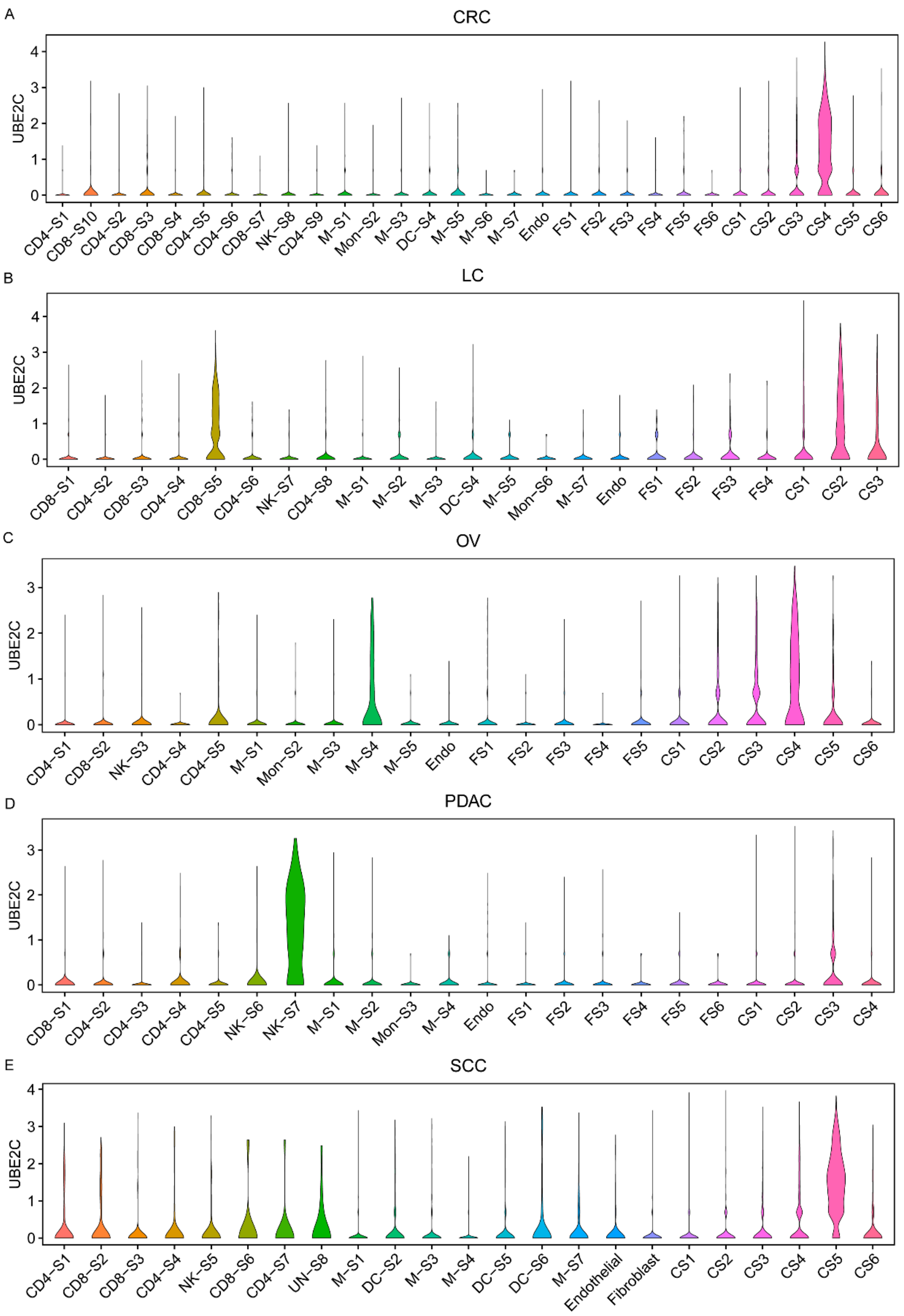

Figure S4 The expression level of *UBE2C* in different cell types in cancer tissues. (A) CRC, Colorectal Cancer; (B) LC, Lung Cancer; (C) OV, Ovarian Cancer; (D) PDAC, Pancreatic ductal adenocarcinoma; (E) SCC, Squamous cell carcinoma; CS represents cancer cells, FS represents fibroblast, CD represents T cells, NK represents natural killer cells, DC represents dendritic cells, Endo represents Endothelial, M represents macrophage and Mon represents monocyte.

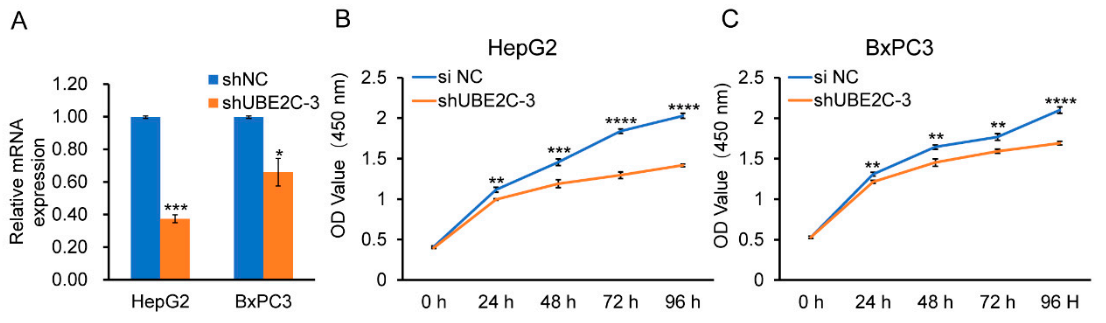

Figure S5 Interference with *UBE2C* significantly inhibits cancer cell proliferation. (A) The expression level of *UBE2C* was detected by qRT-PCR after interference with *UBE2C* and NC in HepG2 and BxPC3 cell lines. Data are representative from three independent experiments. CCK-8 results of HepG2 (B) and BxPC3 (C) cells interfered with *UBE2C* and NC at different time points. Data are representative from at least six independent experiments. The statistical significance was analyzed by Student's t-test (one-tailed) analysis. *: $p < 0.05$; **: $p < 0.01$; ***: $p < 0.001$; ****: $p < 0.0001$.

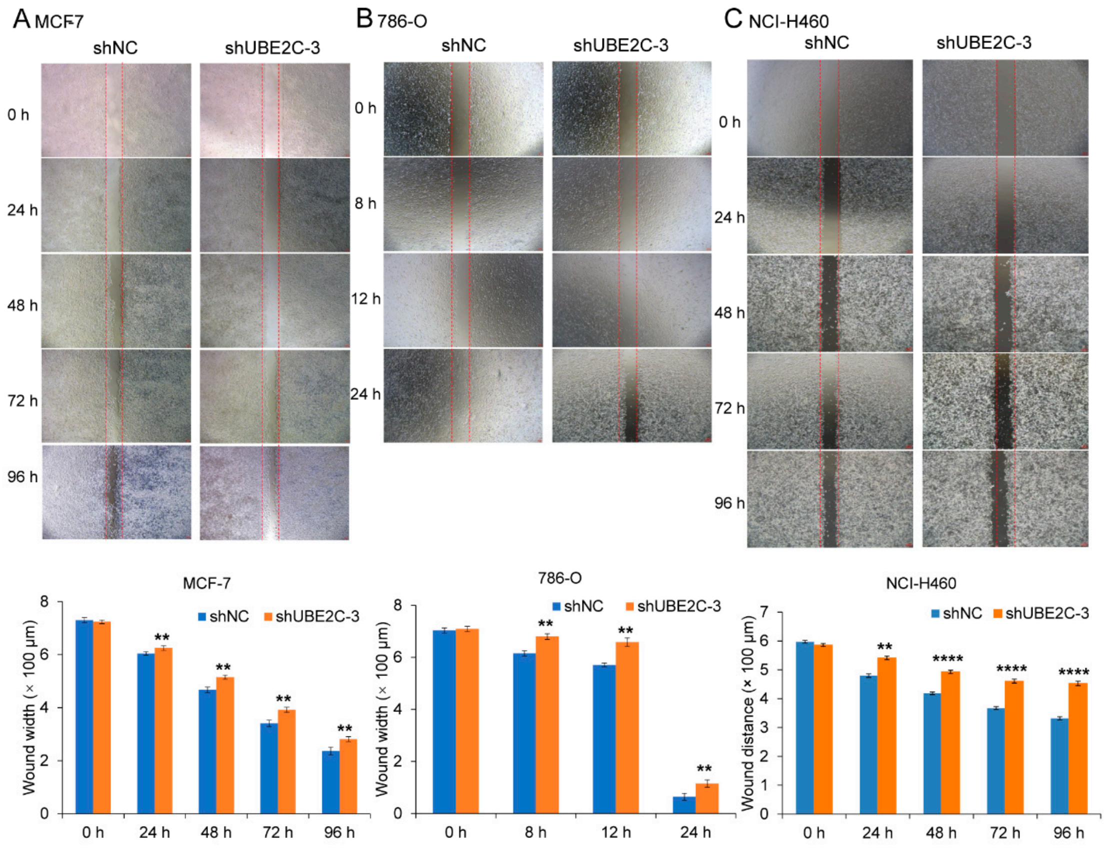

Figure S6 Interference with *UBE2C* significantly inhibited cancer cell migration. (A) MCF-7, (B) 786-O and NCI-H460 (C) cells were interfered with *UBE2C* and then counted the wound width at different time points. The bar graph on the right shows that at each time point, the treatment group and the control group each take at least 9 pictures of the field of view for trace width statistics. Use ImagJ software to calculate the average distribution of 8 straight lines in each image, and the values indicate mean ± SEM. Data are representative from three independent experiments. The statistical significance was analyzed by Student's t-test (one-tailed) analysis. *: $p < 0.05$; **: $p < 0.01$; ***: $p < 0.001$; ****: $p < 0.0001$.

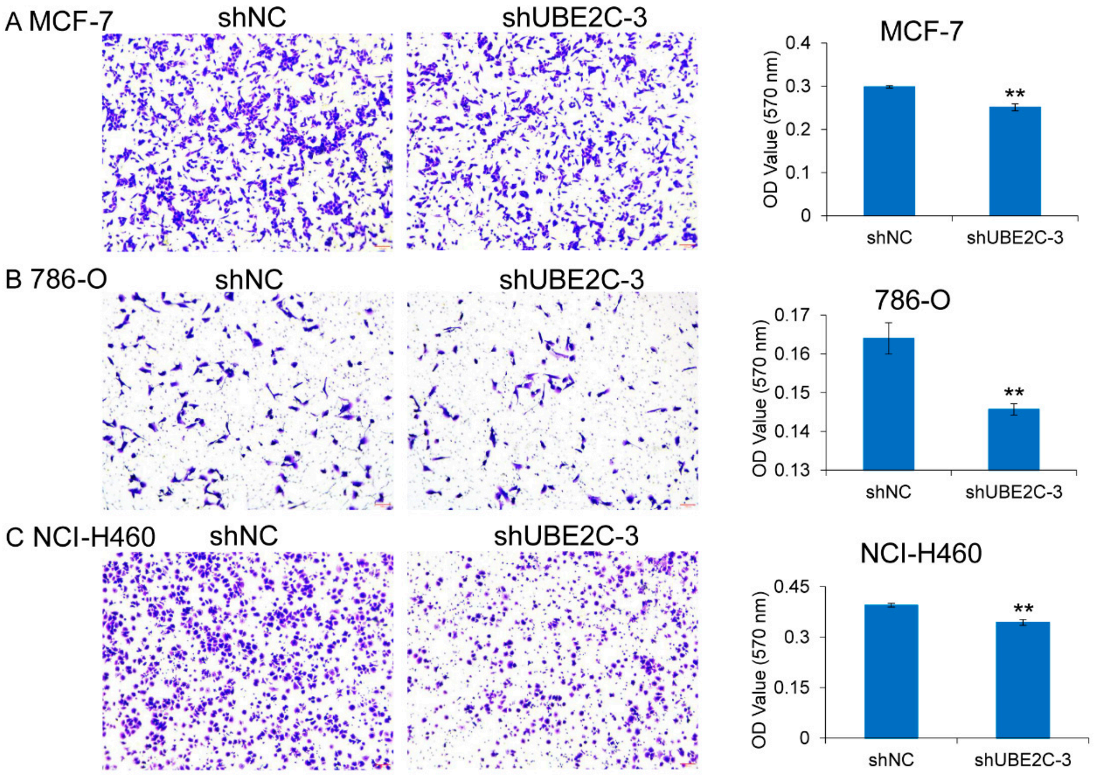

Figure S7 Interfering *UBE2C* could remarkably repressed cancer cell invasion. (A) MCF-7, (B) 786-O and NCI-H460 (C) cells interfere with *UBE2C* and then analyzed the invation cells at 48 h. The bar graph on the right represents the detection of the OD value at 570 nm of crystal violet staining of the treatment group and the control group. The value represents mean ± SEM. Data are representative from three independent experiments. The statistical significance was analyzed by Student's t-test (one-tailed) analysis. **: $p < 0.01$.

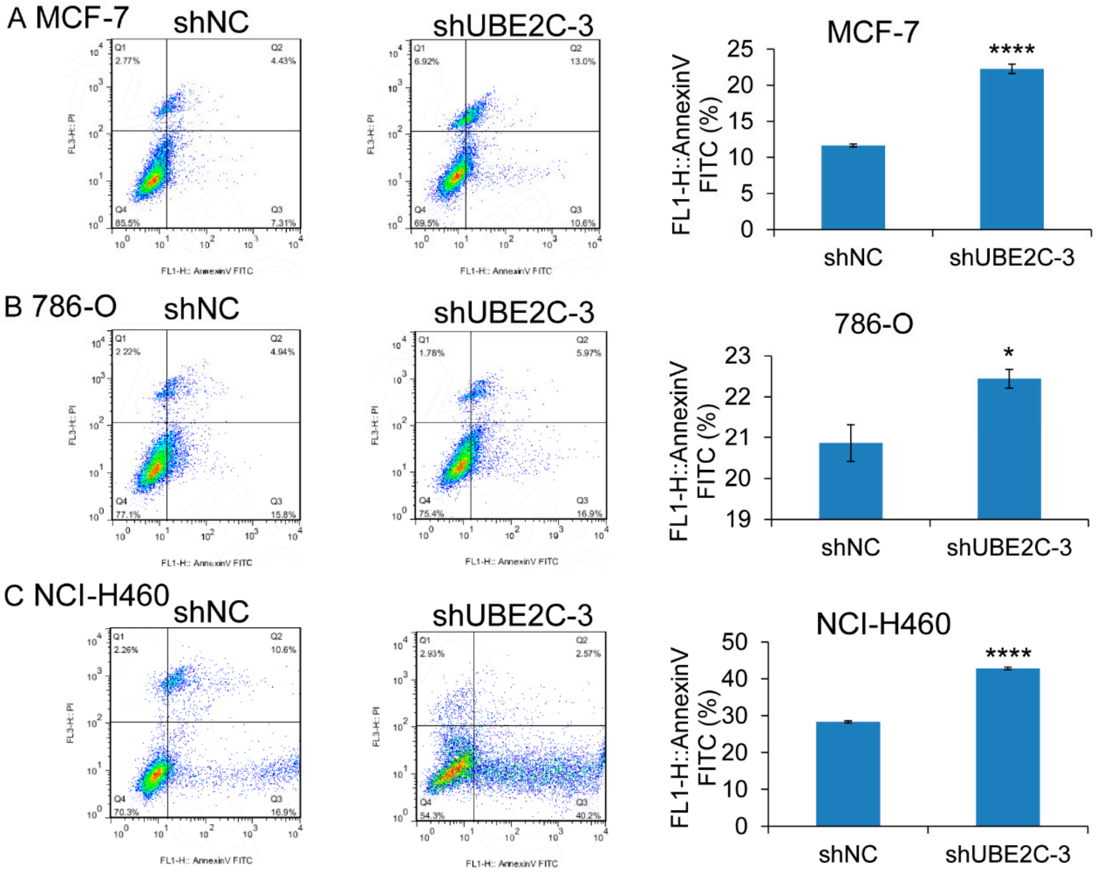

Figure S8 Interference with *UBE2C* significantly promoted cancer cell apoptosis. (A-C) The number of apoptosis cells of (A) MCF-7, (B) 786-O and (C) NCI-H460 cells detected by flow cytometry. The bar graph on the right represents the mean ± SEM of (Q2 + Q3) in each group of samples. Data are representative from three independent experiments. The statistical significance was analyzed by Student's t-test (one-tailed) analysis. *: $p < 0.05$; ****: $p < 0.0001$.

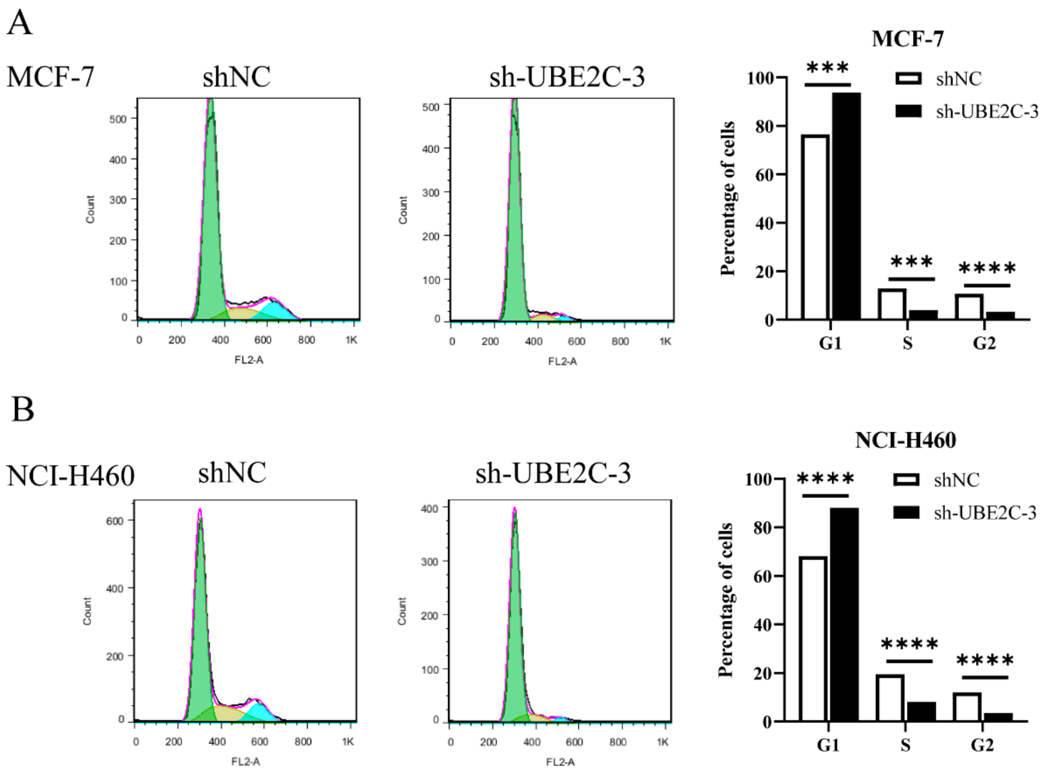

Figure S9 Interference with *UBE2C* significantly induced cell cycle arrest. The cell cycle was detected by flow cytometry in (A) MCF-7 and (B) NCI-H460 cell lines. The bar graph on the right represents the mean ± SEM in each group of samples. Data are representative from three independent experiments. The statistical significance was analyzed by Student's t-test (one-tailed) analysis. ***: $p < 0.001$; ****: $p < 0.0001$.

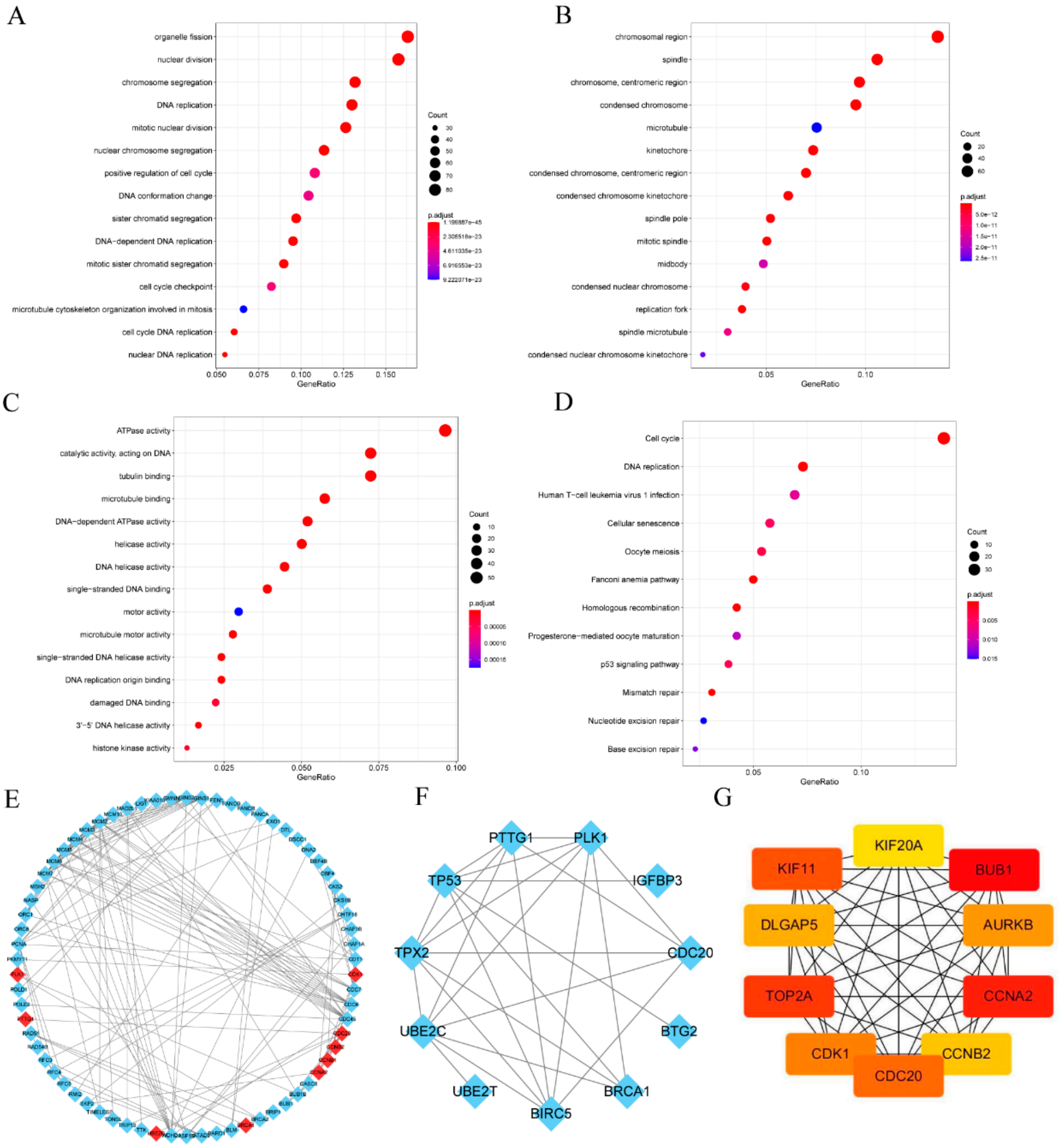

Figure S10 Top 15 of GO terms, Top 12 of KEGG signaling pathways and PPI network of 596 DEGs that regulated by *UBE2C*. **(A-C)** Top 15 pathways of GO_BP (A), GO_CC (B) and GO_MF (C), respectively. Top 12 signaling pathways of KEGG (D); (E,F) Use online software String analysis, among which, select "Experiments" for (E) and “Textming, Experiments, Databases, Co-expression” for (F) at “coactive interaction sources”, and the threshold was set to 0.9; (G) Used Cytoscape software to construct a gene co-expression network, and loaded the plug-in CytoHubba to score the genes in the co-expression network based on the Maximal clique centrality (MCC) method, and screened the 10 hub genes with the highest scores in the co-expression network. The darker the color, the higher the MCC score.

Supplementary Tables

Table S1 Summary of cancer and normal samples analyzed in this study.

Cancer Type	Abbreviation	Number of Tumour Tissue	Number of Normal Tissue
Bladder Urothelial Carcinoma	BLCA	406	19
Breast Invasive Carcinoma	BRCA	1076	113
Cervical Squamous Cell Carcinoma and Endocervical Adenocarcinoma	CESC	291	3
Cholangiocarcinoma	CHOL	36	9
Colon Adenocarcinoma	COAD	438	4
Esophageal Carcinoma	ESCA	161	11
Glioblastoma Multiforme	GBM	153	5
Head and Neck Squamous Cell Carcinoma	HNSC	499	44
Kidney Chromophobe	KICH	64	24
Kidney Renal Clear Cell Carcinoma	KIRC	528	72
Kidney Renal Papillary Cell Carcinoma	KIRP	285	32
Liver Hepatocellular Carcinoma	LIHC	365	50
Lung Adenocarcinoma	LUAD	500	59
Lung Squamous Cell Carcinoma	LUSC	494	49
Pancreatic Adenocarcinoma	PAAD	176	4
Pheochromocytoma and Paraganglioma	PCPG	178	3
Prostate Adenocarcinoma	PRAD	495	52

Rectum Adenocarcinoma	READ	159	10
Sarcoma	SARC	259	2
Skin Cutaneous Melanoma	SKCM	102	1
Stomach Adenocarcinoma	STAD	353	32
Thyroid Carcinoma	THCA	501	58
Thymoma	THYM	118	2
Uterine Corpus Endometrial Carcinoma	UCEC	541	35
SUBTOTAL		8178	693
TOTAL			8871

Table S2 Primers and interfering sequence for target genes used in this study.

Name	Forward sequence	Reverse sequence	Application
KAT2A	TTCCGAGTGGAGAAGGACA	AGCATGGACAGGAATTTGG	qPCR
E2F1	CGCCATCCAGGAAAAGGTGTG	GATGCCCTCAAGGACGTTGG	
UBE2C	CTGCTATCACCCCAACGTGGACA	GACATCATACAGGGCAGACCACT	
GAPDH	ACAACTTTGGTATCGTGGAAGG	GCCATCACGCCACAGTTTC	
pUBE2C-1	CACGCGGAGTAAGACGTGTA	GGCAGAGAGACAGGAACT	ChIP-qPCR
pUBE2C-2	TGTACCCTGCCCGTCTTCCCTT	CCTGGGCAACAGAGCAAGACT	
pUBE2C-3	CGTGTTCTCCGAGTTCCTGT	CCGCATCACTCACCTTTTG	

shKAT2A-1	GATCGCTGAACTTTGTGCAGTACAACTCGAGTTGTACTGCACAAAGTTCAGCTTTTT	AATTAAAAAGCTGAACTTTGTGCAGTACAACTCGAGTTGTACTGCACAAAGTTCAGC	
shKAT2A-2	GATCGGCTACCTACAAGGTCAATTACTCGAGTAATTGACCTTGTAGGTAGCCTTTTT	AATTAAAAAGGCTACCTACAAGGTCAATTACTCGAGTAATTGACCTTGTAGGTAGCC	Interfering *KAT2A*
shKAT2A-3	GATCGCGCATGCCTAAGGAGTATATCTCGAGATATACTCCTTAGGCATGCGCTTTTT	AATTAAAAAGCGCATGCCTAAGGAGTATATCTCGAGATATACTCCTTAGGCATGCGC	
shNC	GATCTTCTCCGAACGTGTCACGTCTCGAGACGTGACACGTTCGGAGAATTTTT	AATTAAAAATTCTCCGAACGTGTCACGTCTCGAGACGTGACACGTTCGGAGAA	
siE2F1-1	GCUAUGAGACCUCACUGAATT	UUCAGUGAGGUCUCAUAGCTT	
siE2F1-2	GCAUCUAUGACAUCACCAATT	UUGGUGAUGUCAUAGAUGCTT	Interfering *E2F1*
siE2F1-3	AGAUGGUUAUGGUGAUCAATT	UUGAUCACCAUAACCAUCUTT	
siNC	UUCUCCGAACGUGUCACGUTT	ACGUGACACGUUCGGAGAATT	
shUBE2C-3	GATCTGTATGATGTCAGGACCATTCCTCGAGGAATGGTCCTGACATCATACATTTTT	AATTAAAAATGTATGATGTCAGGACCATTCCTCGAGGAATGGTCCTGACATCATACA	Interfering *UBE2C*

Table S3 A total of 222 DEGs regulated by of *KAT2A* and *E2F1* and appeared in more than 5 cancer types among 11 different types of cancers at the same time.

Cancer number	Gene number	Gene
10	2	*TONSL CHTF18*
9	7	*PSMC3IP C17orf53 C21orf58 KIF18B TRAIP C16orf59 XRCC2*
8	18	*DBF4B HAUS5 TFAP4 NOP56 CEP131 TSEN54 MYO19 LIG1 RECQL4 LRRC45 ZMYND19 EME1 ORC6 RAD54L MCM7 EZH2 TROAP CDCA3*
7	33	*NR2C2AP BRCA1 PASK DHX34 ILF3 CCDC137 TCOF1 POLE RCCD1 C14orf80 NUP85 GTPBP3 RUVBL1 MXD3 POLD1 PKMYT1 FANCA DSN1 SAPCD2 SPAG5 IQGAP3 DDX39A KNTC1 PSRC1 DONSON ARHGAP11B SPC24 MTBP RFC4 POLE2 AURKB PIDD1 FANCG*
6	61	*LRRC14 TGIF2 RAD51D FBXL19 UBE2O ATXN7L2 KAT2A NAT9 GTF2IRD1 ZNF696 CHAF1A EHMT2 XRCC3 POLR2H PPP2R3B FAM189B SLMO1 NSUN5 AXIN1 E2F1 RRP9 CLCN2 TRMU DDX11 NASP ZWINT TICRR UBE2C CHEK2 WDR5 CAD TAF4 QSOX2 HAUS8 CDC7 CDC45 KIFC1 MGME1 CCDC18 ZGRF1 UBE2T PLK1 LMNB2 CDH24 DNA2 CDT1 WDR90 FANCD2 SUV420H2 IQCC CCNF SAC3D1 ARHGEF39 TCF3 PAM16 RRP1 TRIM65 ANKRD13B C10orf2 CDK5RAP1 FBXO41*
5	101	*HGH1 ZFP41 ZNF707 RRS1 MUTYH RAD9A CCHCR1 C1orf35 LSM4 EXOSC2 KIF22 CPSF1 TRAF2 SLC25A39 MRPL53 DAZAP1 EFTUD2 SMYD5 DHX37 DDX55 TUBG1 SMG5 UCKL1 RPUSD1 TIGD5 ARHGAP39 BOP1 SCRIB PYCRL SDCCAG3 TRAP1 ZNF783 CCDC34 DSCC1 AURKA KIF4A MCM10 KIF23 MCM4 TIMELESS NONO CDC25A RAE1 MIS18A MCM3 NCAPH PABPC1L FANCI KIF15 SLC25A19 NFS1 ARHGEF19 NOP2 DNMT3B CEP72 ESPL1 ARHGAP33 CEP250 FBF1 MDC1 LSM14B SMPD4 PAXIP1 FTSJ3 GIT1 DNMT1 NLE1 HCN3 RAD54B CENPH HELLS ATAD5 H2AFX ALYREF GTSE1 PIF1 DPH7 C19orf48 TOP2A ATAT1 CHAF1B CENPM RCC1 BIRC5 ORC1 NUF2 CDCA5 STMN1 KIF2C SNAPC4 WHSC1 WDR62 CENPK CCNE2 TACC3 ZBTB12 C11orf84 MRGBP PTTG1 WDR4 TK1*
